# Real-world outcomes of photodynamic therapy for HPV-associated lower genital tract lesions: a 2024–2025 gynecology follow-up cohort study

**DOI:** 10.3389/fonc.2026.1810015

**Published:** 2026-07-29

**Authors:** Liqun Wang, Yu Wang, Ziwei Xu, Xuejuan Jiao

**Affiliations:** 1 The First Affiliated Hospital of Bengbu Medical University, Bengbu, China; 2 Bengbu Medical University, Bengbu, China

**Keywords:** 5-aminolevulinic acid, cervical cytology, CIN, HPV-16, HPV-18, human papillomavirus, photodynamic therapy, real-world evidence

## Abstract

**Background:**

Persistent high-risk human papillomavirus (hrHPV) infection drives lower genital tract intraepithelial lesions. 5-aminolevulinic acid photodynamic therapy (5-ALA-PDT) is a minimally invasive, tissue-preserving option, but real-world effectiveness remains variably reported.

**Methods:**

We analyzed de-identified standardized gynecology follow-up forms from 2024–2025 for patients treated with 5-ALA-PDT for HPV-associated lower genital tract disease. Outcomes at 3 months were genotype-specific clearance of HPV-16 and HPV-18 among baseline-positive patients and cytology normalization to negative for intraepithelial lesion or malignancy (NILM) among those with abnormal baseline ThinPrep cytology (TCT). Logistic regression explored predictors of post-treatment HPV clearance.

**Results:**

Overall, 714 patients were included; 635 (88.9%) had complete baseline and follow-up testing plus documented PDT protocol parameters (mean age 45.8 ± 12.6 years). The cervix was the most common treatment site (52.9%); median number of sessions was 4 (IQR 3–6) using 20% 5-ALA. At 3 months, HPV-16 cleared in 162/217 (74.7%), and HPV-18 cleared in 46/61 (75.4%); clearance for other HPV types was 337/566 (59.5%). Among patients with abnormal baseline TCT, 314/329 (95.4%) normalized to NILM. In multivariable analysis, a higher number of baseline HPV types predicted reduced clearance (adjusted OR 0.70, 95% CI 0.50–0.97); age <40 years was associated with clearance only in univariate analysis (OR 1.96, 95% CI 1.17–3.27) but was not significant after adjustment.

**Conclusions:**

In this retrospective routine-care cohort, 5-ALA-PDT was associated with substantial early HPV-16/18 clearance and high short-term cytologic normalization. Multitype HPV infection may be a risk factor and warrant closer surveillance.

## 1 Introduction

Persistent high-risk human papillomavirus (hrHPV) infection is the central etiologic driver of HPV-associated lower genital tract lesions, including cervical intraepithelial neoplasia (CIN), vaginal intraepithelial neoplasia (VaIN), and vulvar intraepithelial neoplasia (VIN), as well as related dysplastic cytology findings (1, 2). Although many low-grade lesions regress spontaneously, persistence of hrHPV, particularly HPV-16 and HPV-18, confers increased risk of progression and recurrence, creating an ongoing need for effective, fertility-sparing, and tissue-preserving therapies alongside structured surveillance (3–6).

Standard management options vary by anatomic site and grade and may include observation, topical agents, ablative techniques, or excisional procedures. However, procedures can be associated with discomfort, scarring, potential reproductive implications, and recurrence remains a common clinical challenge (7). In this context, photodynamic therapy (PDT) has attracted interest as a minimally invasive approach that targets dysplastic or infected epithelium while minimizing structural disruption. PDT, commonly delivered using topical 5-aminolevulinic acid (5-ALA) followed by visible-light irradiation, has emerged as a minimally invasive, tissue-preserving therapy for HPV-associated lesions (8, 9). Mechanistically, 5-ALA is converted to protoporphyrin IX in target tissues, and subsequent light activation generates reactive oxygen species that induce cytotoxic and immunomodulatory effects (8). While clinical studies report favorable short-term clearance and regression outcomes, real-world effectiveness remain heterogenous, and estimates can be difficult to interpret because follow-up is often incomplete outside structured trials (10).

Using routinely collected gynecology follow-up forms from 2024–2025, we evaluated real-world clinical characteristics, PDT protocol parameters, and early post-treatment outcomes. We focused on objective intermediate endpoints used in routine care: clearance of HPV-16/HPV-18 among baseline-positive patients and cytologic normalization to NILM among those with abnormal baseline TCT. We also explored baseline predictors of HPV clearance to support clinical counseling and future study design.

## 2 Methods

### 2.1 Study design, setting, and data source

This observational cohort study used de-identified routine clinical follow-up records of patients treated with photodynamic therapy (PDT) for HPV-associated lower genital tract disease during 2024–2025. At the study site, women with hrHPV-positive test results were managed according to routine gynecologic practice based on HPV genotype, cytology findings, colposcopic impression, and histologic diagnosis when available. In general, women with isolated hrHPV positivity or low-grade abnormalities underwent repeat HPV/cytology surveillance and colposcopic evaluation as clinically indicated, whereas those with more concerning cytologic or colposcopic findings were further evaluated for biopsy and site-directed treatment. In routine practice, 5-ALA-PDT was considered for selected patients with HPV-associated lower genital tract lesions or persistent hrHPV infection, particularly when a tissue-preserving approach was preferred. The decision to offer PDT was made by the treating gynecologist based on lesion site, cytology/colposcopy findings, clinical diagnosis, and patient preference within the local standard-of-care framework. Records were captured using standardized clinical forms that included baseline demographics, HPV genotyping and TCT results, clinical diagnosis, and PDT protocol parameters followed by structured post-treatment assessment. Variables included demographics (age), parity, gravidity, baseline HPV genotyping (HPV-16, HPV-18, and other HPV types), baseline TCT, clinical diagnosis categories, and PDT protocol parameters, including treatment site, number of PDT sessions, drug concentration, drug dose, application time, light intensity, and irradiation time. Follow-up forms recorded repeat HPV testing and TCT. This study used de-identified routine clinical follow-up data. Bengbu Medical University Ethics Committee approved the consent procedures and use of data according to local policies (Ethical Approval No (2025). No. 075).

### 2.2 Study population and analytic cohorts

The source population comprised patients who received 5-ALA photodynamic therapy during 2024–2025 for HPV-associated lower genital tract disease in routine gynecologic practice at the study site. Eligible patients were those with documented hrHPV infection and/or HPV-associated lower genital tract lesions identified on the basis of routine clinical evaluation, including HPV genotyping, TCT, colposcopic assessment, and histopathologic findings when available. In routine practice, 5-ALA-PDT was offered to selected patients with persistent hrHPV infection and/or HPV-associated cervical, vaginal, or vulvar lesions when a tissue-preserving approach was considered clinically appropriate by the treating gynecologist. Patients were excluded from the analytic cohort if baseline or follow-up outcome data were missing or if PDT protocol parameters were not documented. In addition, patients with concurrent conditions that could substantially confound outcome assessment, such as other significant lower genital tract diseases, known immune disorders, or malignant tumors, were not considered eligible to be included in our analysis. All records were used to describe overall data volume and follow-up completeness. For outcome analyses requiring baseline testing and protocol documentation, the primary analytic cohort comprised patients with complete baseline and follow-up testing fields and recorded PDT protocol information, yielding 635 patients (out of a total of 714). Patients’ follow-up data were recorded if the relevant follow-up test result (HPV or TCT) was available. Of the 79 excluded records, 70 lacked follow-up HPV/TCT outcome data, 3 lacked PDT protocol information, and 6 lacked both.

### 2.3 Outcomes

HPV infection duration was defined as the interval, in months, between the date of the first documented positive HPV test and the date of initiation of 5-ALA photodynamic therapy. For patients with more than one HPV genotype detected at baseline, infection multiplicity was defined according to the baseline HPV genotyping result: single-type infection indicated detection of one HPV genotype, whereas multitype infection indicated detection of two or more HPV genotypes. The variable “number of HPV types” was analyzed as the total number of baseline HPV genotypes detected in each patient.

The primary virologic outcomes were genotype-specific clearance of HPV-16 and HPV-18 at 3-month follow-up post-treatment assessment among patients who were baseline positive for the respective genotype and had a recorded follow-up result. Clearance was defined as conversion from baseline positive to follow-up negative for that genotype. The cytologic outcome was normalization of TCT at 3 months among patients with abnormal baseline cytology, defined as conversion to negative for intraepithelial lesion or malignancy (NILM) at the 3-month assessment.

The 3-month time point was selected because it represented the earliest routinely scheduled and most consistently documented post-treatment assessment in the standardized clinical follow-up forms used at our center. We therefore focused on short-term intermediate outcomes that were available with the greatest completeness across the routine-care cohort.

### 2.4 Statistical analysis

Continuous variables were summarized using mean (SD) or median (IQR) depending on distribution; categorical variables were summarized as counts and percentages using available denominators, considering missing follow-ups. Follow-up completeness was assessed by the presence of recorded follow-up HPV/TCT results.

Predictors of post-treatment HPV clearance were evaluated using logistic regression, with HPV clearance at follow-up as the binary outcome. Candidate predictors considered *a priori* included age category, gravidity, parity, HPV infection duration, number of HPV types, infection multiplicity (single vs multiple HPV infection), baseline cytology category, transformation zone type, clinical diagnosis group, and HPV serotype grouping (HPV16/18 vs non-16/18). Prior to multivariable logistic regression, multicollinearity among candidate predictors was assessed using tolerance and variance inflation factor (VIF) statistics, and model calibration was evaluated with the Hosmer–Lemeshow goodness-of-fit test. Variables showing substantial collinearity were not retained together in the final multivariable model. Both univariate and multivariable odds ratios (ORs) with 95% confidence intervals (CIs) were reported.

## 3 Results

### 3.1 Baseline clinical characteristics

The study included 714 participants with baseline information (2024: n=341; 2025: n=373). The mean age was 45.81 ± 12.6 years in all included participants. 635 of patients (88.93%) had complete baseline and post-treatment data and documented PDT protocol information, making the primary analytical cohort. 60.47% of patients were older than 40 years old. Most of them are gravid and parity of 1 or 2 (cumulatively 72.19% and 82.01%, respectively). 45.35% of them were only affected by one serotype of HPV, however, others were affected by at least two concomitant serotypes. The prevalence of HPV-16 and HPV-18 were 34.17% and 9.6%, respectively. However, not mutually exclusive, 89.13% of patients (89.13%) were affected by any other serotypes (other than 16 and 18 serotypes). Baseline TCT results were NILM in 306 (48.18%), LSIL in 167 (26.29%), HSIL in 8 (1.25%), ASCUS in 121 (19.05%), ASC-H in 9 (1.41%). Among those with a transformation zone recorded (n=510), most of the patients had type 3 conversion zone (58.82%, n=300), followed by type 1 (26.86%) and type 2 (14.31%). Among patients with clinical diagnosis, the most common final clinical diagnosis was LSIL (37.87%) and VaIN1 (23.48%) (**Table 1**).

**Table 1:** Baseline characteristics of participants.

Characteristics	Category/level	Mean (SD)/count (percentage %)			
		HPV16 & 18 (n = 270)	Non-HPV16 & 18 (n = 365)	Total (n = 635)	p value
Age	<40 years	119 (44.07%)	132 (36.16%)	251 (39.52%)	0.044^a^
	>40 years	151 (55.93%)	233 (63.84%)	384 (60.47%)	
Gravid	0	39 (14.55%)	40 (10.96%)	79 (12.48%)	0.681 ^a^
	1	75 (27.99%)	97 (26.58%)	172 (27.17%)	
	2	120 (44.78%)	165 (45.21%)	285 (45.02%)	
	3	26 (9.70%)	51 (13.97%)	77 (12.16%)	
	>3	8 (2.99%)	12 (3.29%)	20 (3.15%)	
Parity	0	30 (11.11%)	26 (7.14%)	56 (8.83%)	0.166 ^a^
	1	97 (35.93%)	124 (34.07%)	221 (34.85%)	
	2	125 (46.30%)	174 (47.80%)	299 (47.16%)	
	3	11 (4.07%)	32 (8.79%)	43 (6.78%)	
	>3	7 (2.59%)	8 (2.20%)	15 (2.36%)	
HPV duration (months)		14.43 (17.34)	16.86 (20.6)	15.83 (19.31)	0.109^b^
Single or multiple HPV types	Single	69 (25.55%)	219 (59.51%)	288 (45.35%)	<0.001 ^a^
	Multiple	201 (74.44%)	149 (40.49%)	347 (54.64%)	
Number of HPV types	1	69 (25.84%)	219 (59.51%)	288 (45.35%)	<0.001 ^a^
	2	104 (38.95%)	96 (26.09%)	200 (31.49%)	
	3	45 (16.85%)	31 (8.42%)	76 (11.96%)	
	4	27 (10.11%)	18 (4.89%)	45 (7.08%)	
	5	15 (5.62%)	1 (0.27%)	16 (2.51%)	
	>5	7 (2.62%)	3 (0.82%)	10 (1.57%)	
HPV type	HPV 16	217 (81.27%)	0 (0.00%)	217 (34.17%)	N/A
	HPV 18	61 (22.85%)	0 (0.00%)	61 (9.6%)	
	Other HPV types	201 (75.28%)	365 (100.00%)	566 (89.13%)	
TCT	NILM	139 (51.48%)	167 (45.75%)	306 (48.18%)	0.077 ^a^
	LSIL	56 (20.74%)	111 (30.41%)	167 (26.29%)	
	HSIL	6 (2.22%)	2 (0.55%)	8 (1.25%)	
	ASCUS	53 (19.63%)	68 (18.63%)	121 (19.05%)	
	ASC-H	4 (1.48%)	5 (1.37%)	9 (1.41%)	
	Other	12 (4.44%)	12 (3.29%)	24 (3.77%)	
Conversion type	Type I	59 (27.19%)	78 (26.62%)	137 (26.86%)	0.862 ^a^
	Type II	32 (14.75%)	41 (13.99%)	73 (14.31%)	
	Type III	126 (58.06%)	174 (59.39%)	300 (58.82%)	
Clinical Diagnosis	LSIL	79 (36.07%)	121 (39.16%)	200 (37.87%)	<0.001 ^a^
	HSIL	27 (12.33%)	17 (5.50%)	44 (8.33%)	
	VaIN1	33 (15.07%)	91 (29.45%)	124 (23.48%)	
	VaIN2	32 (14.61%)	23 (7.44%)	55 (10.41%)	
	VaIN3	12 (5.48%)	2 (0.65%)	14 (2.65%)	
	HPV infection	30 (13.70%)	37 (11.97%)	67 (12.68%)	
	Genital warts	6 (2.74%)	18 (5.83%)	24 (4.54%)	

^a^ chi-square test.

^b^ Independent T-test.

### 3.2 Comparison of HPV16/18 vs HPV non-16/18

Baseline characteristics differed modestly between the HPV16/18 and non-HPV16/18 groups. Participants with HPV16/18 were less likely to be >40 years old compared with those with non-HPV16/18 (OR for age >40, HPV16/18 vs non-HPV16/18 = 0.72; 95% CI 0.52–0.99; p=0.044). Gravid and parity distributions were similar between groups (p=0.681 and p=0.166, respectively), and mean HPV duration did not differ (14.43 ± 17.34 vs 16.86 ± 20.60 months; p=0.109). HPV16/18 infections were more often accompanied by multiple HPV types (74.16% vs 40.49%; OR for multiple vs single infection = 4.22; 95% CI 2.99–5.95; p<0.001), and were more likely to involve ≥3 concurrent HPV types (35.21% vs 14.40%; OR for ≥3 types vs 1–2 types = 3.23; 95% CI 2.20–4.74; p<0.001). Baseline cytology (TCT) did not differ significantly between the HPV16/18 and non-HPV16/18 groups (p=0.077). Transformation zone type was comparable (p=0.862). Clinical diagnosis differed significantly (p<0.001): the HPV16/18 group had higher odds of HSIL at baseline (12.33% vs 5.50%; OR for HSIL vs non-HSIL = 2.42; 95% CI 1.28–4.55), and a higher proportion of VaIN3 (5.48% vs 0.65%), whereas the non-HPV16/18 group had more VaIN1 (29.45% vs 15.07%) and genital warts (5.83% vs 2.74%). Percentages are column percentages based on available data for each characteristic.

### 3.3 PDT protocol patterns

PDT protocol characteristics are summarized in **Table 2**. The cervix was the most common treatment site (n=336, 52.91%), followed by the vaginal wall (n=106, 16.69%), vagina (n=106, 16.69%), and vulva (n=27, 4.25%). The number of PDT sessions had a median of 4 (IQR 3–6). 5-ALA was always used at 20% concentration. Drug dose was most frequently 354 mg (n= 436, 69.1%) or 472 mg (n= 150, 23.8%) with median of 354 (IQR 354-472). Application time was mostly 3.0 hours (except for three patients with 3.5 hours and three patients with 4 hours treatment duration). Light intensity was typically 80 mW/cm² (except one with 60, two with 86 and one with 100 mW/cm²), and irradiation time was commonly 25 minutes (23 patients with 20 and 21 patients with 30 minutes of irradiation time).

**Table 2:** PDT protocol characteristics.

Variable	Value
Treatment site, n (%)	Cervix: 336 (52.91%); Vaginal wall: 106 (16.69%); Vagina: 106 (16.69%); Vulva: 27 (4.25%)
Number of PDT sessions	Median 4 (IQR 3–6)
5-ALA concentration	20%
Drug dose, mg	Median 354 (IQR 354–472)
Most common drug doses, n (%)	354 mg: 436 (69.1%); 472 mg: 150 (23.8%)
Application time	Mostly 3.0 h
Light intensity	Typically 80 mW/cm²
Irradiation time	Commonly 25 min

IQR, Interquartile range.

### 3.4 HPV clearance

At the 3-months post-treatment follow-up, among patient with baseline HPV-16–positive patients (n=217), 162 were cleared from HPV-16 by 3 months (74.65%). Among baseline HPV-18–positive patients (n=61), 46 were cleared from HPV-18 by 3 months (75.41%). Among patients with other HPV types (n=566), 337 patients were cleared from all those specific serotypes (59.54%) (**Table 3**).

**Table 3:** HPV clearance by type.

Baseline HPV serology	Clearance	Clearance count	Percent
HPV16 (n=217)	Yes	162	74.65%
	No	55	25.34%
HPV18 (n=61)	Yes	46	75.41%
	No	15	24.59%
Other HPV types (n=566)	Yes	337	59.54%
	No	229	40.45%

HPV, Human Papillomavirus.

### 3.5 Cytology outcomes

Normalization from abnormal baseline TCT finding to NILM occurred in 314/329 (95.44%) 3-months post-treatment (**Table 4**).

**Table 4:** Baseline and follow-up TCT results.

Before treatment cytology	After treatment cytology					Total
	NILM	LSIL	HSIL	ASCUS	ASC-H	
NILM	306	0	0	0	0	306
LSIL	161	3	0	3	0	167
HSIL	6	0	0	2	0	8
ASCUS	114	4	0	3	0	121
ASC-H	9	0	0	0	0	9
Other	24	0	0	0	0	24
Total	620	7	0	8	0	635

NILM, Negative for Intraepithelial Lesion or Malignancy, LSIL, Low-grade, Squamous Intraepithelial Lesion, HSIL, High-grade Squamous Intraepithelial Lesion, ASCUS (ASC-US), Atypical Squamous Cells of Undetermined Significance, ASC-H, Atypical Squamous Cells-cannot exclude HSIL.

### 3.6 Factors affecting the HPV clearance after PDT

In univariate logistic regression using the post-treatment HPV clearance endpoint, age <40 years (vs >40) was associated with higher odds of clearance (OR 1.96, 95% CI 1.174–3.272), and a higher number of HPV types was associated with lower odds of clearance (OR 0.719, 95% CI 0.583–0.885). Single HPV infection (vs multiple) and transformation zone Type I (vs Type II/III) were also associated with clearance in univariate analysis. In multivariable regression, the number of HPV types remained independently associated with clearance (adjusted OR 0.697, 95% CI 0.500–0.971), while other covariates did not retain statistical significance. Multicollinearity diagnostics showed no evidence of problematic collinearity among predictors included in the adjusted model (all VIFs < 2.519]; all tolerances > 0.397). The final multivariable logistic regression model showed acceptable fit on the Hosmer–Lemeshow goodness-of-fit test (χ² = 9.147, df = 8, p = 0.33) (**Table 5**).

**Table 5:** Factors associated with HPV clearance.

Factor	Univariate logistic regression*		Multivariable logistic regression**	
	Univariate OR (95% CI)	Univariate p value	Multivariate OR (95% CI)	Multivariate p value
Age <40 (Ref >40)	1.96 (1.174 – 3.272)	0.01	1.791 (0.890 – 3.601)	0.102
Gravidity	0.916 (0.723 – 1.162)	0.472		
Parity	1.027 (0.758 – 1.392)	0.862		
HPV duration	0.993 (0.983 – 1.003)	0.183		
Number of HPV types	0.719 (0.583 – 0.885)	0.002	0.697 (0.500 – 0.971)	0.033
Single HPV type (Ref: Multiple HPV types)	1.805 (1.099 – 2.964)	0.02	1.101 (0.454 – 2.254)	0.978
TCT (NILM) (Ref: ASCUS/LSIL)	1.184 (0.663 – 2.115)	0.569		
Conversion zone (Type I) (Ref: Type II/III)	1.824 (1.006 – 3.307)	0.048	1.23 (0.57 – 2.655)	0.597
Clinical Diagnosis	0.923 (0.768 – 1.108)	0.388		
HPV serotype (HPV 16&18) (Ref: Non-16&18)	1.427 (0.583 – 3.491)	0.436		

HPV, Human Papillomavirus, TCT, ThinPrep Cytologic Test, NILM, Negative for Intraepithelial Lesion or Malignancy.

*Candidate variables evaluated in the univariate logistic regression analyses were selected based on clinical relevance.

**Variables entered into the multivariable logistic regression model were selected based on univariate screening and clinical relevance.

Collinearity diagnostics showed no problematic multicollinearity among predictors retained in the final model. The final adjusted model showed acceptable fit according to the Hosmer–Lemeshow goodness-of-fit test.

## 4 Discussion

In this real-world gynecology cohort treated with topical 5-ALA-PDT for HPV-associated lower genital tract disease, we observed substantial early virologic response and strong short-term cytologic improvement. At 3 months, genotype-specific clearance was highest for HPV-16 and HPV-18 (74.65% and 75.41%, respectively), while clearance for other HPV types was lower (59.54%). In parallel, among patients with abnormal baseline ThinPrep cytology, normalization to NILM occurred in 95.44% at the 3-month assessment. Together, these findings support the clinical utility of 5-ALA-PDT as a tissue-preserving approach that can achieve meaningful early HPV and cytologic endpoints in routine care settings (8).

Our clearance rates for HPV-16/18 at 3 months compare favorably with previously published clinical series and reinforce the notion that treatment response depends on the underlying genotype mix and clinical context. In a prospective study of hrHPV-positive women without cervical lesions, Cang et al. reported hrHPV clearance of 56.1% at 3 months and 68.1% at 6 months after three sessions of ALA-PDT, with higher clearance among HPV-16/18 infections and lower clearance in multitype infections (11). Similarly, Han et al. described high overall HPV clearance after ALA-PDT in CIN/VaIN patients, increasing from 72.5% at 6 months to 85.7% at 12 months (9). These studies align with our observation that HPV-16/18 demonstrate robust early response, while “other” HPV types show comparatively lower clearance. A rational clinical interpretation is that early “test-of-response” is more informative when stratified by genotype and multiplicity, rather than relying solely on pooled hrHPV endpoints.

Mechanistically, 5-ALA-PDT is thought to act through selective accumulation of protoporphyrin IX and subsequent photoactivation, leading to reactive oxygen species generation, with both cytotoxic and local immune-modulatory effects (12, 13). A contemporary synthesis of ALA-mediated photodynamic applications in lower genital tract disease highlights that these combined effects may contribute to viral clearance and regression of HPV-associated epithelial abnormalities, while preserving tissue architecture (8). The high rate of early cytologic normalization we observed is consistent with this field-directed therapeutic rationale, particularly for patients in whom fertility preservation, avoidance of cervical scarring, or minimization of anatomic disruption is prioritized (7, 14).

An important contribution of our analysis is the identification of baseline infection multiplicity as a predictor of virologic persistence after PDT. In multivariable regression, a higher number of baseline HPV types independently predicted lower odds of post-treatment HPV clearance (adjusted OR 0.697). This finding is consistent with broader evidence that multiple high-risk HPV infections represent a higher-risk biological milieu with less favorable post-treatment trajectories (15). In a systematic review and meta-analysis, Cassani et al. found that multiple hrHPV infections at the time of standard HSIL treatment conferred a small but significant increase in risk of persistence/recurrence, supporting the value of multiplicity as a risk stratifier in follow-up planning (16). Our data extend that concept to the PDT setting and suggest that multiplicity may be a practical counseling variable. Patients with multitype infections may require additional treatment sessions, closer surveillance, or a longer interval before confidently declaring durable clearance.

These results also have implications for the post-treatment surveillance strategy. HPV persistence after treatment is known to decline over time, but estimates vary widely by modality, HPV type, and the timing of testing. Hoffman et al. summarized that median HPV persistence decreases from about 27% at 3 months to approximately 15% at 12 months following CIN treatment, emphasizing both the dynamic nature of clearance and the importance of standardized follow-up windows for interpretation (17). Risk-based follow-up frameworks further emphasize HPV testing as a cornerstone of post-treatment management, with intensity tailored to estimated CIN3+ risk rather than a single “one-size-fits-all” algorithm (7). In practice, our findings support using early post-PDT HPV testing as a meaningful intermediate endpoint, while recognizing that persistence at 3 months, especially in multitype infections, should prompt structured repeat testing and careful colposcopic assessment consistent with contemporary risk-based guidance.

Several features of this study strengthen the relevance of the findings to real-world care. First, the cohort is comparatively large for PDT outcomes research and reflects routine clinical documentation rather than highly selected trial populations, improving ecological validity. Second, we reported genotype-specific outcomes for HPV-16 and HPV-18 rather than only pooled hrHPV positivity, which enhances clinical interpretability given the differing oncogenic risks and natural histories across types. Third, standardized recording of PDT protocol parameters (drug concentration, dose, application time, light intensity, irradiation time) and baseline covariates enabled exploratory modeling of predictors of clearance, generating hypotheses for protocol optimization and future prospective work (10).

Limitations should be considered when interpreting the magnitude and generalizability of effect estimates. The most important limitation is incomplete follow-up, which was substantially lower in 2025 and may introduce selection bias if follow-up availability is correlated with symptom burden, perceived improvement, socioeconomic access, or clinician-driven triage. Additionally, outcomes were based on routine clinical records rather than a controlled protocol with uniform visit timing; therefore, “3-month” assessments may vary in actual interval, potentially diluting precision. Longer-term follow-up data were not available in a sufficiently complete and standardized manner for the present analysis. The study design is observational and lacks an internal comparator group (e.g., standard excisional/ablative management or observation), which prevents causal attribution and limits the ability to distinguish treatment effect from spontaneous clearance, especially for lower-grade disease. The observed effect estimates might differ in a controlled comparative design or with longer follow-up, because spontaneous clearance, delayed response, and recurrence cannot be fully captured in a single-arm 3-month routine-care analysis. We also focused on intermediate endpoints (HPV clearance and cytology normalization) rather than histologic regression endpoints across all diagnoses, and “other HPV types” were analyzed as a heterogeneous category, obscuring potential differences between specific non-16/18 high-risk genotypes. Finally, although we explored predictors of clearance, residual confounding is likely, and some clinically relevant factors (e.g., smoking, immunosuppression, vaccination status, sexual behavior, and adherence to post-treatment recommendations) were not available in the extracted dataset. In addition, it should be noted that the retrospective nature of our study hindered us from performing a robust subgroup analysis due to limited sample size in some subgroups and our exploratory finding’s should be interpreted cautiously. More investigations in future studies are needed to explore differences in the efficacy of 5-ALA-PDT across different patient subgroups. Despite these limitations, the overall pattern of results is clinically coherent and supported by existing literature.

## 5 Conclusions

5-ALA-PDT can achieve high early HPV-16/18 clearance and substantial short-term cytologic normalization in routine practice, while infection multiplicity appears to be a consistent marker of reduced response and may justify more intensive surveillance. Future research should prioritize prospective designs with standardized follow-up, genotype-resolved endpoints beyond HPV-16/18, and clinically anchored outcomes such as histologic regression and recurrence over 12–24 months. Integrating risk-based follow-up algorithms and evaluating protocol refinements (number of PDT sessions, dose-response, and field coverage) may further optimize effectiveness for patients at higher risk of persistence, particularly those with multitype infections.

## Data Availability

The raw data supporting the conclusions of this article will be made available by the authors, without undue reservation.
